# Phthalate Esters in Aquatic Ecosystems: A Multiscale Threat from Molecular Disruption to Ecological Risks

**DOI:** 10.3390/toxics14020185

**Published:** 2026-02-23

**Authors:** Zhicheng Sun, Marriya Sultan, Jian Han, Chunsheng Liu, Yanbo Ma

**Affiliations:** 1 Chongqing Institute of Green and Intelligent Technology, Chinese Academy of Sciences, Chongqing 400714, China; 2 Chongqing School, University of Chinese Academy of Sciences, Chongqing 400714, China; 3 School of Public Health, Chongqing Medical University, Chongqing 400016, China; 4 State Key Laboratory of Freshwater Ecology and Biotechnology, Institute of Hydrobiology, Chinese Academy of Sciences, Wuhan 430072, China; 5 MOE Key Laboratory of Groundwater Quality and Health, School of Environmental Studies, China University of Geosciences, Wuhan 430074, China

**Keywords:** plastic additives, trophic dilution, epigenetic reprogramming, toxicity cascade, quantitative adverse outcome pathway

## Abstract

Phthalate esters (PAEs), ubiquitous plastic additives, have emerged as persistent contaminants in aquatic ecosystems, yet their propagation from molecular initiating events to ecosystem-level collapse remains poorly integrated. This review synthesizes current knowledge on the source-to-sink dynamics of PAEs, revealing a critical paradox in their bioaccumulation patterns: unlike classical persistent organic pollutants, high molecular weight PAEs exhibit distinct trophic dilution rather than biomagnification along food webs, driven by metabolic biotransformation in higher trophic organisms. Despite this dilution, PAEs trigger a bottom-up toxicity cascade. Driven by molecular initiating events, PAEs induce a range of adverse effects at the individual level, including immunotoxicity, neurotoxicity, endocrine disruption, metabolic dysfunction, and trans-trophic oxidative stress. Crucially, prolonged exposure drives epigenetic reprogramming, which reduces reproductive output, thereby threatening long-term population recruitment. These individual and population deficits could escalate into higher ecological consequences, specifically by diminishing benthic biological control over phytoplankton, dampening energy transfer efficiency, and simplifying community structure, thereby posing a potential threat to primary productivity and aquatic ecosystem sustainability. Despite recent advances, critical knowledge gaps remain, particularly regarding their cascading impacts on ecosystem services, as well as synergistic interactions between PAEs and other contaminants. In order to validate laboratory results with actual ecological risk assessments, future research should incorporate multi-scale models and quantitative adverse outcome Pathways as well as their synergistic interactions between PAEs and other contaminants, and advanced in vitro systems such as organoids. Resolving these issues is essential to reducing the risks that PAEs pose to aquatic environments.

## 1. Introduction

Phthalate esters (PAEs) are the primary plasticizers used in a wide range of plastic products, constituting 87% of global plasticizer consumption [1]. China accounts for 42% of this total usage, with 90% of its domestic consumption dedicated to polyvinyl chloride (PVC) production [2]. The global production of phthalate esters (PAEs) has continued to rise, currently estimated at approximately 8 million tons annually [3]. The most widely used PAEs include dimethyl phthalate (DMP), diethyl phthalate (DEP), dibutyl phthalate (DBP), butyl benzyl phthalate (BBP), di-cyclohexyl phthalate (DCHP), di(2-ethylhexyl) phthalate (DEHP), diisobutyl phthalate (DIBP), diisononyl phthalate (DINP), diisodecyl phthalate (DiDP), di-n-hexyl phthalate (DnHP), and di-n-octyl phthalate (DnOP). PAEs pose serious risks to aquatic life and human health because of their easy leaching into aquatic ecosystems along the production-use-disposal continuum as a result of their non-covalent integration into polyolefin plastics [4]. Some high-risk PAEs have been designated as persistent organic pollutants (POPs) by the Stockholm Convention, and China has also designated them as priority substances under its New Pollutant Control Action, which calls for stricter monitoring and emission reductions across consumer goods and environmental media.

PAEs are widely found in aquatic ecosystems across the globe and exhibit notable regional variation, according to numerous studies. Single-compound PAE readings frequently surpass 100 µg/L in Eastern Europe. For instance, DMP and DEHP in Polish surface waters reached 210 µg/L, whereas DEHP in Lithuanian rivers reached 159 µg/L [5]. The PAE profiles of the various Asian areas varied significantly. In South Korea, near-shore waters next to residential areas have total PAE values higher than 0.2 µg/L [1], whereas the average total PAE concentration in rivers in northern Vietnam was 125 µg/L [6]. According to data from important river basins in China, Taihu Lake surface waters had 13.0 μg/L Σ_6_PAEs, with DBP (1.6 μg/L) and DEHP (1.3 μg/L) predominating [7]. In the Yangtze River Basin, the total concentrations of Σ_15_PAE varied from 2.7 to 81.8 µg/L, with DBP peaking at 70.3 µg/L [8,9]. Similar gradients were found in marine systems. The north-western Mediterranean Gulf of Marseille’s total PAEs reached 1.3 µg/L [10], while concentrations in tropical regions of the western Pacific were significantly lower [11,12].

PAEs dissolve, volatilize, adsorb sediment, degrade, and bioaccumulate after being discharged into aquatic environments. These mechanisms control the migration, dispersion, and transformation of PAEs throughout environmental compartments, which ultimately results in a variety of harmful impacts on aquatic biota. In aquatic organisms, PAEs interfered with immunity, growth, development, and reproduction. PAEs primarily inhibit algal growth by decreasing pigment content and causing oxidative stress, which is typified by increased levels of reactive oxygen species (ROS) and malondialdehyde as well as increased superoxide dismutase (SOD) and catalase (CAT) activity [13,14]. In water fleas, *Daphnia magna* (*D. magna*), exposure to PAE reduces antioxidant capability, encourages lipid buildup, and inhibits fatty acid intake and catabolism [15]. Studies on fish have shown that DBP causes oxidative damage by inducing hepatic SOD and glutathione peroxidase (GPx) surges in common carp *Cyprinus carpio* (*C. carpio*) [16]. By changing gene expression and dysregulating sex hormones, PAEs interfere with zebrafish (*Danio rerio*) reproduction, which ultimately hinders gametogenesis, lowers reproductive success, and causes transgenerational damage [17,18]. Furthermore, PAEs compromise zebrafish immune responses by inducing splenic ROS buildup, inhibiting antioxidant enzymes (SOD, CAT), impairing mitochondrial function, and disrupting lipid metabolism [19]. In the end, these organism-level impacts jeopardized ecosystem stability and functional resilience by lowering population densities, interfering with trophic energy transmission, and simplifying community structures [20].

Few studies examine the combined impact of PAEs with co-occurring microplastics, heavy metals, or pharmaceutical residues, while most ecological risk assessments of PAEs still focus on individual compounds [21]. Significant knowledge gaps exist about long-term ecological concerns, including transgenerational effects and community cascading reactions triggered by environmentally relevant dosages in ecosystems, as a result of the majority of toxicological studies using high-dose, short-term exposure models [22,23]. In addition to revolutionizing traditional risk-assessment frameworks, the combination of multi-omics and AI-driven big-data analytics also offers new mechanistic insights into the intricate exposure routes and multi-target toxicity of PAEs [24,25,26]. In light of this, this review methodically summarizes the most recent findings about the environmental destiny and multi-level harmful effects of PAEs in aquatic environments, with a focus on: (1) aquatic habitats’ sources and exposure paths; (2) toxicological processes in communities, populations, and individuals; and (3) important difficulties in determining PAE toxicity in intricate situations, as well as potential research topics. The purpose of this review is to guide PAE research and stimulate advanced methods for ecological risk assessment.

## 2. Meta-Analysis

**Figure 1:**
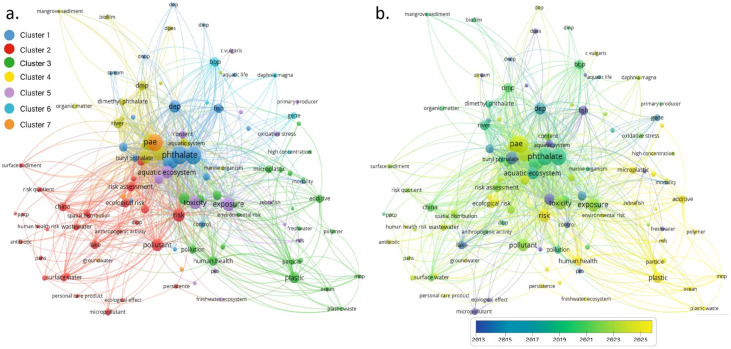
Meta-analysis of studies related to PAEs in aquatic ecosystems based on co-occurrence of keywords using VOSviewer software (version 1.6.17). Varying node size indicates the frequency of keyword appearance across selected publications. Curved lines between nodes represent co-occurrence within the same publication, while the proximity between nodes reflects the strength of their co-occurrence. (**a**) Network visualization showing thematic clustering into seven distinct groups. (**b**) Overlay visualization illustrating the temporal distribution of keywords from 2013 to 2025 based on average publication year.

A bibliometric approach using VOSviewer was adopted, following procedures applied in previous work [27]. In this comprehensive meta-analysis, data from 132 scholarly articles focusing on environmental fate, ecotoxicity, and health implications of phthalate esters in aquatic ecosystems were meticulously examined. The literature spanned a temporal range from 2013 to 2025 and was retrieved primarily from Scopus, with supplementary sources including Web of Science, Google Scholar, and Sciwheel to ensure exhaustive coverage. The search strategy employed a combination of key terms such as “phthalate,” “aquatic ecosystem,” “freshwater pollution,” “plastic additive,” “ecotoxicity,” and “risk assessment.” All selected publications were archived in Sciwheel, exported in RIS format, and subsequently imported into VOSviewer for bibliometric mapping and clustering analysis. VOSviewer’s full-counting algorithm was applied to extract keyword co-occurrence data, with a minimum threshold of eight occurrences set for inclusion. Vague and repetitive keywords were manually removed to enhance thematic clarity. The resulting network visualization comprised 3539 links with a total link strength of 42,379, revealing seven distinct clusters that reflect the structural composition of the literature (Figure 1a). Nodes represent individual keywords, with font size proportional to their frequency and edge thickness indicating the strength of co-occurrence. Curvilinear connections denote intra-publication keyword relationships, while spatial proximity between nodes reflects cross-study thematic convergence.

Cluster 1 (29 items) addressed a wide range of environmental and public health concerns of PAEs, with a strong emphasis on their ecological and toxicological impacts. Cluster 2 (24 items) consolidated studies on PAEs-associated additives and their ecotoxicological and endocrine-disrupting effects, featuring keywords like microplastic, plastic polymer, PVC, endocrine disruptor, and human health. Cluster 3 (22 items) encompassed key terms related to the ecological risks and toxicological profiles of various PAE compounds in aquatic organisms. It included specific chemical entities of PAEs, indicating a focus on biological endpoints and organism-level impacts, particularly in seawater, stream, and sediment samples. Cluster 4 (17 items) centered on the environmental dynamics and fate of PAEs within aquatic systems, emphasizing their accumulation, distribution, and transformation across various ecological compartments. Cluster 5 (15 items) focused on the toxicological effects and ecological implications of PAE exposure within freshwater ecosystems. Cluster 6 (6 items) concentrated on the toxicological mechanistic and bioindicator relevance of PAEs. Cluster 7 (3 items) focused on the intersection of water pollution and health risk, and examined the leaching behavior of PAEs from plastic materials or industrial sources into subsurface water systems.

The overlay visualization (Figure 1b) delineates the chronological progression of keyword usage across the dataset. Early research from 2013 to 2016 predominantly addressed the environmental distribution and sedimentary accumulation of phthalates, particularly in marine and mangrove ecosystems. Between 2016 and 2019, the focus shifted toward ecotoxicological endpoints, with increased attention to oxidative stress, growth inhibition, and endocrine disruption in aquatic organisms. The period from 2019 to 2022 saw a surge in studies on specific phthalate compounds such as DEHP and BBP, including their bioaccumulation in fish and invertebrates. Most recent research, spanning 2022 to 2025, has expanded into risk assessment frameworks, integrating molecular biomarkers, genetic toxicity, and human health implications, particularly in freshwater and groundwater systems.

The geographical distribution of the analyzed literature exhibits significant spatial heterogeneity. A predominant number of field monitoring and case studies originate from Asia and Europe. Specifically, research in Asia is heavily concentrated in China, with a focus on major catchments such as the Yangtze River and Taihu Lake, as well as in Northern Vietnam. Similarly, European studies are largely centered on the Northwestern Mediterranean and Rhône River catchments. Regarding research objects, investigations in these data-rich regions typically employ a multimedia framework that encompasses surface water, sediments, and diverse biota ranging from plankton to commercial fish. In contrast, empirical data from regions including West Asia and East Africa remain comparatively scarce. Notably, studies in these underrepresented areas have primarily prioritized abiotic matrices, such as water and sediment, or wastewater effluents. Consequently, comprehensive assessments of biological burdens within local aquatic food webs are lacking in these regions. This disparity in both geographical coverage and research targets highlights a critical gap in global data comparability.

## 3. Migration and Exposure Pathways of PAEs in Aquatic Systems

### 3.1. Sources and Migration

**Figure 2:**
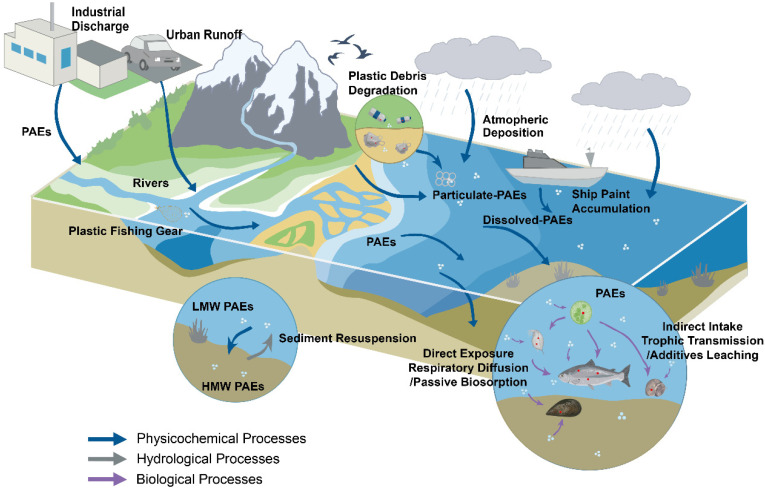
Environmental sources and migration pathways of PAEs in aquatic ecosystems.

Due to their extensive use, hydrophobicity, and semi-volatility, PAEs have complicated aquatic environmental origins. The primary input pathways include industrial effluent discharge [28,29], deterioration of plastic waste [30,31,32], leaks from boats and aquaculture equipment [33], runoff from cities [34], as well as atmospheric deposition [35] (Figure 2). Among these, industrial activities remain the most significant source of PAEs. The accumulated sludge ΣPAEs at a Taiwanese municipal wastewater treatment plant that treats wastewater from the plastic, paint, and textile dyeing industries was 3.24 × 10^4^ µg/kg dw [36]. An advanced biological plant and artificial wetlands in Konya discharged 1997 kg/d of PAEs, around 90% of the buildup in waste sludge [37]. A wastewater treatment plant in Iran that collects effluent from the plastic and petrochemical sectors was a persistent source of downstream river contamination, with an influent DEHP mean concentration of 30.1 µg/L [38]. Furthermore, when discarded plastics were exposed to UV light, thermal oxidation, and microbiological activity, significant amounts of PAEs were generated. According to studies, the annual DEHP release flow from ancient plastics is 50–80% higher than that from new plastics [39]. Aquaculture facilities and ship anti-fouling paint were the main sources of PAEs in marine environments. Kaohsiung Port sediments had ΣPAEs of up to 8.7 × 10^3^ ng/g dw, which was far higher than nearby offshore values [40]. Sediment ΣPAEs peaked at 54.3 µg/g dw (dry weight) in Hong Kong aquaculture cage locations, surpassing neighboring non-aquaculture sites by more than 10 times, suggesting persistent leaching from PVC cages and feed pipes [41]. Another important factor is urban mobility, as urban roads release 0.23 to 1.9 kg of tire wear particles annually per person, which carry PAEs into surface waterways through stormwater runoff [42]. Notably, 34–42% of the yearly PAE inputs to coastal ecosystems come from atmospheric dry and wet deposition. High-molecular-weight (HMW) species primarily transfer via air–water volatilization within this flow, while low-molecular-weight (LMW) PAE homologues primarily undergo dry deposition [35].

In a variety of environmental interfaces, PAEs show notable migration and redistribution traits (Figure 2). Its multiphase partitioning behavior between water, particulates, and organic materials is controlled by its log K_ow_ (octanol–water partition coefficient) and K_oc_ (organic carbon–water partition coefficient). Along seven Yangtze River transects, DEHP constituted 38.9% of Σ_14_PAEs in sediment and correlated strongly with total organic carbon, indicating preferential adsorption of HMW PAEs onto organic carbon [43]. DIBP/DnBP/DEHP and total organic carbon exhibited comparable positive associations in surface sediments from the Bohai and Yellow Seas [44]. PAEs moved from the dissolved to the particulate phase in estuaries as a result of increased salt-induced precipitation brought on by growing salinity. Salinity significantly enhances the partitioning of PAEs from the dissolved phase to particulates. Evidence from 30 estuarine systems reveals that for every 5‰ increase in salinity, the particle–water partition coefficient increases by an average of 0.3 log units. This amplification can approach 0.6 log units in high-salinity areas, such as the Lion Gulf in the Mediterranean, which greatly accelerates the migration of PAEs from the dissolved phase to suspended particles [45]. Microplastics (MPs) were unique particle vectors that served as endogenous leaching sources in addition to adsorbing PAEs from ambient matrices. The greatest partition coefficient for DEHP in polystyrene particles was 10^3^ L/kg, and aging treatment increased the subsequent desorption rates by 20–80% [39,46]. Research in Kumamoto, Japan, indicated that a single storm may wash MP-bound PAEs from road dust to riverbed sediment, where they would form a secondary reservoir for subsequent resuspension. And MP abundance also showed a substantial correlation with measured phthalates [47]. Significant redistribution between dissolved and particulate forms occurred as a result of particulate PAEs being net exported seaward through estuarine tidal outlets during the flood season [48]. As a result, organic matter and PAE-laden particles were consistently carried from sources to downstream and coastal waters by runoff and tides. For instance, about 5.28–19.52 tons of Σ_24_ PAEs were input into the Bohai Sea annually by surface runoff, with the vast majority being in suspended particle form, with substantial contributions during the wet season [28].

Sediments serve as both key sources and sinks of PAEs in regulated or semi-enclosed water basins (Figure 2). DBP and DEHP deposition rates as high as 1.81–17.6 µg/m^2^·d were detected using sediment traps in Poland’s Nielisz reservoir, suggesting that particle PAEs quickly settle in stagnant water conditions and continuously replenish surface sediments [49]. Summer–autumn surface-sediment Σ_16_PAE values in Hangzhou Bay’s semi-enclosed tidal systems ranged from 118 to 5888 µg/kg dw. According to partitioning experiments, medium molecular weight PAEs (DIBP and DnBP) stayed in close to dynamic equilibrium, whereas HMW PAEs (DEHP and DnOP) tended to partition and settle from the aqueous phase into the sediment phase [50]. Sediment, however, was not PAEs’ permanent sink. In anoxic sediments, hypoxic circumstances greatly prolonged the half-life of HMW PAEs by inhibiting their microbial degradation. Because of this inhibition, PAEs continue to diffuse upward through the sediment–water interface, maintaining sharp concentration gradients between sedimentary porewater and overlying water [20]. Similarly, sediment resuspension is directly caused by physical disturbances resulting from maritime activities. Monitoring data from France’s Gulf of Marseille demonstrated that bottom water ΣPAE concentrations rose from 130 to 1330 ng/L from late summer to early autumn [51]. During high hydrological events, flow acceleration, tidal shear, and vessel wakes dramatically increased particle resuspension and lateral movement, magnifying PAE rerelease fluxes, according to a meta-analysis of over 30 estuarine and coastal datasets [45]. Furthermore, biota are also important dynamic reservoirs for PAEs. Several studies have shown that fish and benthic organisms accumulate 10^2^–10^3^ L/kg of PAEs [36]. Marine mussels quickly absorbed long-chain PAEs, reaching a biological concentration factor (BCF) of 3.5 × 10^3^ L/kg within 24 h, according to isotope tracing [52]. Soft corals were also shown to achieve a BCF of 7.9 × 10^3^ L/kg for medium-chain DBP in recent microcosm investigations [53]. These results showed that the spatiotemporal heterogeneity of PAE ecological risks is governed by a multimedia fate network that is composed of water, sediments, and biota.

### 3.2. Exposure Pathways of Aquatic Organisms to PAEs

#### 3.2.1. Direct Exposure

Strong hydrophobicity allowed dissolved PAEs to reach aquatic creatures by respiratory diffusion in aquatic animals and passive biosorption in algae (Figure 2). Through quick passive processes, microalgae effectively absorbed organic contaminants until the concentrations in the aqueous phase and the adsorbed quantities reached equilibrium [54]. The main method by which PAEs passed across cellular membranes was passive diffusion. Increased hydrophobicity resulted in higher degrees of permeability and accumulation [55,56]. Notably, the amount and nature of extracellular polymeric substances (EPS) released by microalgae altered contaminant biosorption and had a substantial impact on PAE adsorption [57]. Gills were the main interfaces for the uptake of lipophilic pollutants in fish, especially during the development of the embryo and larva [58]. The fish Green chromide, *Etroplus suratensis*, showed noticeably greater DEHP loads and oxidative stress in its gills than in its muscle following a 7-day exposure to DEHP, indicating that the gills were the primary exposure portals [59]. The gills of three fish species from the Italian coast of Campania had far higher PAE concentrations than other organs, according to analyses, with DIBP reaching up to 1061 ng/g dw [60]. The gills of the filter-feeding hard-shelled mussel *Mytilus coruscus* serve as both primary PAE interception interfaces and dual respiratory filtration surfaces, with tissue accumulation hierarchies arranged as follows: gill > gonad > muscle tissue [52].

#### 3.2.2. Indirect Intake

Additionally, trophic transmission and the leaching of additives from consumed MPs might expose aquatic animals to PAEs indirectly (Figure 2). According to studies, MPs act as chronic chemical sources by continuously leaking PAEs and other intrinsic additives [61]. Feeding experiments showed that ingesting polyethylene MPs containing PAE dramatically increases fish hepatic PAE levels [62]. An average of 92 MP particles carrying PAEs were found in the stomachs of yellowfin tuna, *Thunnus albacares*, while ΣPAE concentrations in red muscular tissue were 7.3 × 10^4^ ng/g ww [63]. DEHP levels reached 2.3 µg/g dw, with a statistically significant positive correlation found between the number of stomach MP particles and ΣPAEs concentrations from five Pacific shark species [64]. Interestingly, even when biomagnification factors (BMFs) are less than 1, ongoing trophic transfer may result in a notable accumulation of PAEs in species at higher trophic levels. DEHP accumulated in algae and daphnids in a tri-trophic microcosm and was entirely transmitted to fish, increasing its concentration to 3.4 × 10^2^ ng/g lw in fish liver [65]. Additionally, because of their higher aqueous solubility and relatively slow metabolic rates, LMW PAEs showed stronger bioaccumulation in zooplankton and low-trophic-level fish, exhibiting a characteristic “LMW-high burden” pattern [66,67,68,69].

## 4. Accumulation and Transfer of PAEs in Aquatic Organisms

### 4.1. Bioaccumulation in Primary Trophic Levels

Algae are important entry points for contaminants into aquatic food webs since they are primary producers. PAEs build up in lower-trophic-level creatures due to their strong hydrophobicity and lipophilicity, which makes it easier for them to be biomagnified in higher-trophic-level organisms later on [70]. Sea lettuce, *Ulva lactuca*, a marine alga, quickly absorbed six PAEs, according to laboratory exposure tests, and showed a consistent improvement in absorption capability throughout the course of the testing period. Additionally, algae preferentially accumulated PAEs with HMW and octanol–water partition coefficients (log K_ow_) [71]. Phytoplankton have a substantially greater potential to bioaccumulate long-chain PAEs than higher trophic organisms, according to a three-year study carried out in the waters of Taiwan’s Kaohsiung Harbor. The highest concentration of DINP in yellowfin tuna muscle from the same seas was 1.5 times lower than the DINP concentration in phytoplankton, which was 1.11 × 10^5^ ng/g dw [32,63]. The Ligurian shore also showed this pattern, with algae continuously exhibiting superior accumulation of long-chain phthalate esters, especially DINP and DEHP [55,56]. The main mechanism for PAE enrichment in algae is passive adsorption, driven by hydrophobic interactions. However, recent comprehensive reviews suggest that the uptake process in broader aquatic plants is more complex than simple diffusion [72], highlighting that in aquatic macrophytes (e.g., *Lemna minor*), PAE uptake can involve energy-dependent pathways assisted by carrier proteins. Specifically, molecular docking evidence indicates that non-specific lipid transfer proteins (*nsLTPs*) facilitate the transmembrane transport and intracellular translocation of PAEs via apoplastic and symplastic pathways [72]. Although compared to anthropogenic emissions, algal biosynthesis makes up a very small portion of environmental PAE pollution, its mechanisms and ecological ramifications demand more research [73]. Additionally, research shows that co-exposure to DBP and microfiber MPs changed the extracellular polymeric substance (EPS) composition on the surface of algal cells. This change improved the absorption and bioaccumulation of PAEs in algal communities by raising the DBP partition coefficient between algal biomass and microplastics by 20–40% [57]. Together, adsorption, endogenous synthesis, and pollutant interactions determine the starting concentrations that can be transferred to higher trophic levels and dictate the spatiotemporal variability of PAE accumulation in phytoplankton.

The main way that zooplankton accumulate PAEs is by consuming tainted algae and suspended particles. According to a study conducted in Marseille Bay, France, the mean concentration of Σ_7_PAE in plankton was 7230 ng/g dw, with smaller size classes bearing almost half of the burden. These organisms have a bioconcentration factor (BCF) of about 10^3^ and preferentially collect extremely hydrophobic congeners (DEHP and DnBP) [69]. Mediterranean investigations provided additional evidence for this trend, as field tests of biological samples showed that zooplankton had maximum Σ_7_PAE concentrations of 4.58 µg/g dw, with the highest enrichment levels found in smaller individuals [69,74]. Similarly, an investigation of the whole basin of Taihu Lake, China, showed that zooplankton had a significant accumulation of PAE, with Σ_6_PAE concentrations reaching 72.7 µg/g dw. The greatest bioaccumulation factor (log BAF) value of 3.7 further emphasizes freshwater zooplankton as a key bioaccumulation mechanism for PAEs in aquatic environments, with DEHP found in 100% of samples [67]. Further evidence that zooplankton are important PAE carriers comes from long-term monitoring in the North Pacific, where concentrations show spatiotemporal fluctuations and were positively connected with community biomass [75]. All of these results demonstrate that zooplankton, especially tiny and lipid-rich species, are important vectors in aquatic food webs and effective PAE bioaccumulators.

### 4.2. Bioaccumulation in High-Trophic-Level Organisms

During nutritional intake throughout the food chain, PAEs may bioaccumulate in aquatic creatures as trophic levels rise (Table 1). Large fish usually have ΣPAE values between 10^2^ and 10^3^ µg/kg ww. In Poyang Lake, China, for example, an analysis of 11 edible fish species showed ΣPAE values ranging from 118.6 to 819.8 µg/kg ww, with DEHP and DBP together accounting for over 90% of all pollutants. Benthic species showed noticeably greater loads than pelagic migratory fish [76]. A median ΣPAE concentration of 0.14 µg/g dw was found in measurements of 130 muscle samples from commercial fish in five estuaries, with DEP and DEHP collectively accounting for over 70% of the total content [77]. Open-ocean habitats continue to accumulate PAE. In certain selected samples, the median DEHP contents of Mediterranean sardines *Sardina pilchardus* were greater than those of other esters and exceeded the 10 ng/g ww limit of quantification [78]. ΣPAE levels in silk shark muscle from the open waters of the Indian Ocean ranged from 5.4 to 34.6 mg/kg, with DEHP accounting for half of the contaminant burden [79].

Filter-feeding bivalves and crustaceans had lower ΣPAE loads than organisms at higher trophic levels. Consistent with their high metabolic capacity, DEHP in fish is rapidly metabolized in the liver into monoesters (e.g., MEHP), resulting in higher concentrations of metabolites in bile and liver compared to muscle tissues [3]. The concentration of Σ_7_PAEs in Mediterranean mussels, *M. galloprovincialis*, from the coast of Lazio, Italy, ranged from 57 to 131 ng/g ww, with DEHP leading in terms of both detection frequency and concentration [80]. Green mussels in northern Vietnam had an average of 524 ng/g ww of Σ_13_PAEs, ranging from 35.7 to 1747 ng/g ww [82]. Both wild and raft-cultured mussels in Galicia, Spain, had Σ_5_PAEs at 37–7146 ng/g dw. The DEHP accumulation in raft-cultured stocks was especially significant, with DEHP predominating in raft stocks [83]. DEHP values of 0.45–0.70 mg/kg ww were found in commercial shrimp market monitoring. Although these concentrations were below the EU regulation limit of 1.5 mg/kg, they nevertheless represented a significant human exposure pathway [84]. In a related study, analysis of 341 seafood samples from China’s Hangzhou Bay found a mean Σ_8_PAE concentration of 238 ng/g ww. The order of decline was crabs > fish > shrimps > shellfish [81].

### 4.3. Trophic Transfer in the Aquatic Food Chain

Numerous parameters, including dietary sources, respiratory exchange, excretion rates, metabolic transformation, and chemical structural characteristics, affect the biomagnification potential of PAEs [68,85]. HMW congeners (DEHP, DnOP, and DINP) experienced trophic dilution, exhibiting reduced in lipid-normalized concentrations at higher trophic positions, while LMW PAEs (DMP, DEP, DIBP, DBP, and BBP) remained stable in lipid-normalized concentrations across trophic levels, according to bioconcentration models [68]. According to physiological studies, HMW PAEs mainly accumulate through nutritional intake in high-trophic-level fish, while LMW ones are mostly absorbed and eliminated by gills. Nevertheless, their biomagnification factor (BMF) typically stays below 1, limiting substantial buildup along the food chain, because of their quick gastrointestinal hydrolysis and effective metabolic conversion [68]. A quantitative analysis of the data presented in Table 1 further elucidates these patterns and challenges the assumption of uniform biomagnification. Although phytoplankton act as a major reservoir for PAEs, with DEHP concentrations reaching as high as 39,118 ng/g ww, this burden does not linearly transfer to higher trophic levels. For instance, predatory fish such as *Thunnus albacares* exhibit significantly lower burdens (DEHP 33.60–495.23 ng/g ww in meat), representing an approximately 100-fold decrease from phytoplankton to tuna. This marked reduction supports the hypothesis of trophic dilution for HMW PAEs in aquatic food webs. Furthermore, the tissue-specific accumulation patterns highlight the significant role of metabolic clearance. In species such as yellowfin tuna *T. albacares* and red mullet *M. barbatus*, gill tissues show significantly higher concentrations of LMW PAEs (e.g., DMP up to 1273 ng/g ww) compared to muscle tissue (e.g., DMP up to 275 ng/g ww). This discrepancy suggests that although LMW PAEs are readily absorbed through respiratory surfaces, they undergo rapid biotransformation before accumulating in muscle, thereby limiting systemic biomagnification, as often predicted by conventional risk models.

In the Chinese Songhua River (freshwater) and Bohai Bay (marine), all PAEs exhibited trophic dilution, with freshwater exhibiting less dilution than seawater [86]. Interestingly, in both systems, PAEs’ BCF increments were significantly smaller than PCBs’ for every unit rise in logK_ow_, indicating that high-trophic-level metabolic activities successfully offset the bioaccumulation potential anticipated by lipophilicity [70]. In summary, according to available data, there is no discernible biomagnification of PAEs in aquatic food webs, and high-trophic-level creatures exhibit substantial trophic dilution. Nonetheless, there are still significant unanswered questions about the bioaccumulation behavior of PAE metabolites in food chains, which is a top research priority that needs to be thoroughly examined.

## 5. Multilevel Toxic Effects of PAEs

### 5.1. Toxicity at the Individual Level

#### 5.1.1. Oxidative Stress

**Figure 3:**
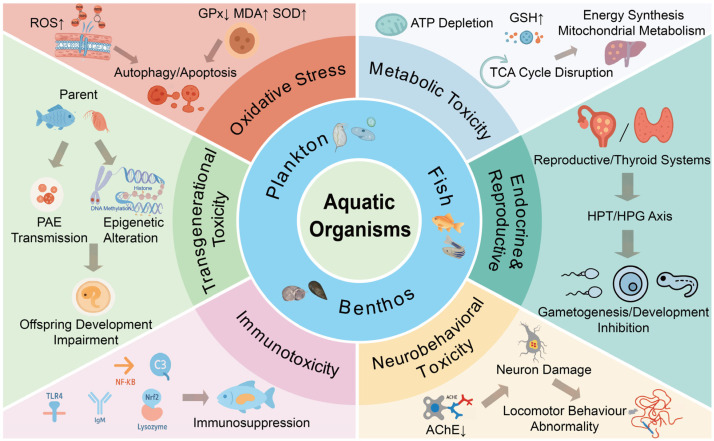
Multidimensional toxicological mechanisms of PAEs on aquatic organisms at the individual level.

In aquatic species, phthalates (PAEs) cause a variety of toxicities, including oxidative stress, energy metabolism problems, endocrine disruption, neurotoxicity, immunotoxicity, and impairments that last over generations (Table 2, Figure 3). Recent data demonstrates that PAEs cause transtrophic oxidative stress by compromising antioxidant defenses and producing excessive amounts of reactive oxygen species (ROS). PAEs quickly upset the oxidative equilibrium of microalgae, which are the principal photosynthetic producers. After 96 h of exposure to 20–200 µg/L DMP and DEP, the marine diatom *Phaeodactylum tricornutum* displayed markedly higher intracellular ROS levels and higher amounts of malondialdehyde (MDA), a crucial lipid peroxidation indicator [87]. Similarly, after being exposed to 2–10 mg/L DEHP for 5 days, the freshwater green alga *Chlorella vulgaris* showed increased hydrogen peroxide H_2_O_2_ and MDA buildup, along with diminished glutathione peroxidase (GPx) and superoxide dismutase (SOD) activities [88]. After 48 h of exposure to 0.001–1 mg/L BBP, the rotifer *Brachionus plicatilis* showed rapid oxidative stress responses at the zooplankton level, which were marked by dose-dependent increases in MDA levels and a notable upregulation of antioxidant enzymes [89]. Furthermore, exposure to 100 µg/L DBP and BBP caused water fleas, *D. magna*, to produce too many ROS, leading to an imbalance in its antioxidant system and disturbing its metabolism [90]. A schematic overview of these individual-level toxicity pathways is provided in Figure 3.

When filter-feeding bivalves were exposed to 201 µg/L mixed PAEs for seven days, hard-shelled mussel *M. coruscus* showed a distinctive biphasic pattern of SOD and catalase (CAT) activities, along with a notable increase in glutathione reductase activity [52]. Likewise, after 48 h of exposure to 50 µg/L, the Manila clam *Ruditapes philippinarum* showed increased ROS levels and lipid peroxidation in its gill tissues [91]. After being subjected to DBP, higher-trophic-level crustaceans, including the swimming crab *Portunus trituberculatus*, showed decreased glutathione concentration and increased glutathione GST activity, heat shock protein expression, and MDA levels [92]. Across taxa, investigations of fish species showed consistent patterns of oxidative damage. After acute 24-h exposure to 100–500 µg/L DEHP, goldfish *Carassius auratus* displayed gill oxidative stress, exhibiting compensatory antioxidant enzyme activation, ROS overproduction, and GPx imbalance. Moreover, purine metabolism dysregulation was identified by integrated omics analysis as a major factor in this species’ buildup of ROS [93]. After 28 days of exposure to 500–1000 µg/L DBP, adult zebrafish, *Danio rerio*, showed reduced SOD and CAT activity, increased MDA levels, and testicular histopathology with impaired spermatogenesis [94]. Notably, excessive ROS accumulation induced by PAEs can trigger autophagy as an adaptive cellular defense mechanism, as illustrated in Figure 3. Mechanistic studies on aquatic organisms, such as common carp, *C. carpio*, have demonstrated that DEHP exposure leads to a rapid ROS burst, which subsequently inhibits the PI3K/AKT/mTOR signaling pathway and upregulates autophagy-related genes (e.g., *beclin-1*, *lc3b*) [120]. In this context, autophagy initially functions as a cytoprotective process (mitophagy) to degrade ROS-damaged mitochondria and oxidized proteins, thereby maintaining cellular homeostasis. However, if the chemical stress exceeds the cell’s compensatory capacity, this autophagic flux may shift from protective to destructive, ultimately contributing to autophagic cell death or apoptosis. Together, these results demonstrate that PAEs consistently upset aquatic organisms’ oxidative equilibrium via a single toxicity route that includes ROS overproduction, a breakdown in antioxidant defense, and the resulting lipid peroxidation at all trophic levels.

#### 5.1.2. Energy and Metabolic Disorders

In both freshwater green algae (*C. vulgaris*) and marine diatoms (*P. tricornutum*), exposure to 10–5000 µg/L DEP and DMP for 96 h dramatically lowers chlorophyll content and alters mitochondrial membrane potential, which inhibits photosynthesis and energy metabolism and ultimately hinders growth [87]. Similarly, after being exposed to 600 mg/L DMP for seven days, freshwater angiosperms, *Lemna minor*, and *Spirodela polyrhiza* showed lower growth rates, decreased photosynthetic pigment content and photochemical efficiency, significantly upsetting the photosynthetic-carbon metabolic balance [13]. Chronic exposure to 100 µg/L DBP or BBP (alone or co-exposed with nano-CuO) in water fleas, *D. magna*, resulted in a considerable reduction in important TCA cycle intermediates (e.g., oxoglutaric acid), which disturbed energy metabolism. Additionally, untargeted metabolomics showed that aminoacyl-tRNA biosynthesis and arginine biosynthesis pathways were disrupted (with downregulated metabolites), while glutathione metabolism was modulated with enhanced enzymatic activity as a stress response [90]. After 48 h of exposure to 0.01–6 mg/L BBP, marine rotifers (*B. plicatilis*) showed signs of ovarian-cell mitochondrial damage, which hindered oxidative phosphorylation and ATP synthesis. Growth and reproductive deficits resulted from these impacts, which were accompanied by worsened lipid peroxidation, decreased glutathione (GSH) levels, and disturbed energy substrate balance [89].

Following exposure to 2–20 µg/L DMP/DBP/DEHP for seven days, the hard-shelled mussel *M. coruscus* showed increased generation of ROS, persistent elevation of GSH, and depletion of energy metabolites [52]. After 14 days of exposure to 4–324 µg/L DEHP, Mediterranean mussels *M. galloprovincialis* had biphasic responses in glucose, glycogen, and amino acid levels, exhibiting high-concentration inhibition and low-concentration stimulation [96]. MPs significantly increased the bioavailability of DEHP, hindering the recovery of SOD activity in hard-shelled mussel *M.coruscus* and continuously decreasing TCA cycle intermediates, which prevented physiological recovery and resulted in energy depletion [95]. White leg shrimp *Penaeus vannamei* were exposed to 100 µg/L DEHP for 21 days, which caused lipid and amino acid metabolism to be disturbed. This resulted in purine metabolic dysfunction and inflammatory reactions [97]. Across a variety of fish species, PAEs regularly cause metabolic disruptions. After 14 days of exposure to 10–400 µg/L DEHP, the liver of African catfish *Clarias gariepinus* displayed biphasic peroxisome proliferator-activated receptors (PPAR) modulation along with disturbed lipid oxidation and storage pathways [98]. Zebrafish exposed to 3 mg/kg DEHP and overfed for 60 days showed increased energy surplus and obesity risk. Moreover, intestinal transcriptomic analysis implicated a PPAR-mediated lipid metabolic network in this process [100]. After being subjected to 0.42–42 µg/L DINP for 21 days, female zebrafish experienced endocannabinoid system (ECS) imbalance and hepatic steatosis. Mechanistic research verified that DINP modulated the ECS-PPAR axis to increase hunger and hepatic lipogenesis [99]. Dietary exposure to 15–1500 μg/(kg·d) for gilthead seabream (*S. aurata*) DINP inhibits the action of testicular fatty acid amide hydrolase and dramatically lowers plasma 11-ketotestosterone levels [101]. Exposure to 0.1–0.5 mg/L DEHP for 56 days induced elevated levels of alanine transaminase (ALT), aspartate transaminase (AST), triglycerides, and serum glucose in yellowhead catfish *Pelteobagrus fulvidraco* [102].

#### 5.1.3. Disruption of Reproductive and Thyroid Endocrine Functions

Numerous studies have shown that PAEs seriously impair aquatic creatures’ endocrine systems by interfering with nuclear receptor signaling pathways and changing vital hormone metabolism processes (Figure 3). Research on the hypothalamic–pituitary–thyroid (HPT) axis showed that PAEs interfered with thyroid homeostasis at several stages of development. Thyroid-specific genes such as thyroglobulin (*tg*), deiodinase 2 (*dio2*), and transthyretin (*ttr*) were activated when exposed to low-dose DBP (10 µg/L) or DIBP (50 µg/L), which also raised T3 and T4 levels [103]. Furthermore, zebrafish larvae exposed to 1–100 µg/L DINP and its metabolites for 5 days showed markedly increased levels of total thyroxine (TT4), decreased locomotor activity, and changed expression of thyroid and neurodevelopmental genes [104]. After 21 days of exposure to 0.3–300 µg/L DEHP, adult male zebrafish showed a significant decrease in total triiodothyronine (TT3) and an increase in thyroid-stimulating hormone (TSH). Additionally, the expression of the key thyroid hormone deiodinase genes *dio1* and *dio3a* was significantly upregulated, while that of *dio2* was downregulated [121]. Growth impairment and increased expression of thyroid-related genes were seen in Japanese medaka, *Oryzias latipes*, embryos exposed to 3.3–33 µg/L DEHP [105].

Due to the persistent evidence of interference with the hypothalamic–pituitary–gonadal (HPG) axis, the reproductive system becomes the principal target of PAE toxicity. In male zebrafish, long-term exposure to DEHP (10–100 µg/L) changed the transcription and DNA methylation of steroidogenic genes (*cyp17a1*, *hsd17b3*, and *cyp19a1a*), which decreased the production of sex hormones and hindered spermatogenesis [17]. Long-term exposure to DEP/DINP/DEHP in female zebrafish resulted in a reduced gonadosomatic index (GSI), ovarian atrophy, and reproductive failure [106]. From the zebrafish embryonic stage to sexual maturity, chronic exposure to DBP (4.9–43.8 μg/L) significantly decreased female fecundity, as well as the levels of testosterone (T) in males and estradiol (E2) in females. Additionally, it suppressed the expression of the hepatic vitellogenin (*vtg*) gene and downregulated important genes related to the HPG axis, exhibiting typical dual anti-estrogenic and anti-androgenic effects [107]. Similarly, after 60 days of exposure to DEHP (1–100 μg/L), female koi carp *Cyprinus rubrofuscus* showed oocyte shrinkage, changed sex hormone profiles, decreased GSI, and markedly reduced embryo fertilization and hatching rates [108]. Additionally, DEHP exposure (10–400 μg/L) induced concentration-dependent responses in African sharptooth catfish (*C. gariepinus*), including elevated expression of *vtg*, estrogen receptor, and aromatase genes, along with enhanced synthesis of E_2_ and T. These changes were accompanied by ovarian atresia in females and testicular degeneration in males [98].

#### 5.1.4. Neurotoxicity and Behavioral Toxicity

The neurotoxic effects of PAEs were mostly observed in aquatic creatures (Figure 3). Acute DBP exposure (0.5–2 mg/L) in primary consumers caused a marked inhibition of acetylcholinesterase (AChE) activity in water fleas (*D. magna*) [122]. Exposure to LMW PAE mixtures (0.1–10 µg/L) for 48 h disrupted the amino acid and energy metabolism pathways in water fleas (*D. magna*). Metabolomic analysis showed that glutamine-GABA synthesis, choline metabolism, and tRNA aminoacylation were all repressed, indicating altered neurotransmitter synthesis [109]. Choline and GABA were among the neuroactive metabolites that were considerably decreased in hard-shelled mussels (*M. coruscus*) after 14 days of exposure to DEHP or DBP (100 µg/L) [95]. The cholinergic (*ache*) and dopaminergic (*dat*, *th*, *drd1b*) genes were changed in zebrafish embryos exposed to DEHP (0.1–500 µg/L) for 96 h, resulting in hyperactive/inhibited movement throughout light–dark cycles. In order to validate PAE interference with dopamine signaling, prolonged treatment until 7 days post-fertilization (dpf) further decreased the expression of *drd1b* and *th*, increased AChE activity, and disturbed circadian rhythms [110,111]. Glutathione imbalance and neuronal chromatin condensation were developed in adult zebrafish exposed to DEHP (4–324 µg/L) for 96 h, which hindered spatial learning and increased aggressiveness [112]. Furthermore, the expression profile of genes linked to p53 signaling, neurodevelopment, and neurotransduction pathways was changed when juvenile zebrafish were exposed to MEHP (7.4–74 µg/L), an active metabolite of PAEs, for 28 days. Brain tissue showed significant oxidative stress and cellular apoptosis, which eventually resulted in decreased swimming activity, aberrant social behavior, and inhibited locomotor capacity [113].

#### 5.1.5. Immunotoxicity

One important endpoint for PAE toxicity is frequently the immune system (Figure 3). The immune response gene nuclear factor-k-gene binding (*nf-κB)* was markedly increased and Heat shock protein 70 (*hsp70*) expression was downregulated in high-dose groups of *M. galloprovincialis* after 21 days of exposure to DEHP (4–324 µg/L). Meanwhile, the expression of the non-specific lysozyme gene Goose-type lysozymes in *Mytilus galloprovincialis* (*MgGLYZ*) showed a U-shaped trend, while its antioxidant enzyme activity followed an inverted U-shaped pattern, indicating an induced innate immune and cytoprotective response to PAEs in mussels [96]. Exposure to DEHP changed the hepatopancreatic pro-phenoloxidase (proPO) system in the Japanese mud crab, *Macrophthalmus japonicus*. The transcript levels of proPO, phenoloxidase (PO), and peroxinectin (PE) were elevated on the first day but declined precipitously by day seven. In contrast, the expression of serpin and trypsin remained significantly elevated throughout the exposure period [114]. After 28 days of exposure to DEHP (1–5 mg/L), zebrafish showed markedly elevated serum complement component 3 (C3) levels and upregulated transcriptional expression of C3 responses [19]. Long-term immunomodulatory effects were produced by LMW PAEs. For example, zebrafish exposed to environmentally appropriate DBP concentrations (2 µg/L) during embryonic life developed immune–inflammatory responses that continued into adulthood, most likely as a result of disruption of the hypothalamus–pituitary–adrenal axis. This imbalance was further worsened by persistent inflammatory cell and cytokine activation [115]. Immunoglobulin m alpha (*igm-α*) and myeloid differentiation primary response 88 (*myd88*) genes were significantly upregulated in the hepatic tissue of Indian carp (*Labeo catla*) after 30 days of chronic DEP exposure (1.6 mg/L). At the same time, the *myd88* gene were significantly upregulated in brain tissue [116].

#### 5.1.6. Transgenerational Toxicity

According to recent research, gametes or embryonic secretions can directly transmit accumulated PAEs from parents to their children, negatively impacting the development of the offspring (Figure 3). Exposure to 1 µg/L DEHP in F0 water fleas, *D. magna*, resulted in its buildup in ovaries and embryos throughout generations. This was accompanied by increased ROS, ongoing dysregulation of genes linked to reproduction, and a marked decrease in survival and net reproductive rates in the F0 and F1 generations [23]. Similarly, PAEs, which are mainly formed via maternal yolk transfer, were found in the yolk and albumen of unhatched loggerhead sea turtle, *Caretta caretta*, eggs, indicating possible developmental harm to offspring [117]. In addition to directly transferring pollutants, PAEs may alter parental germline epigenetic marks to control gene expression across generations. After six days of exposure to 30 µM MEHP, zebrafish embryos showed 410 differentially methylated regions (DMRs), which were significantly enriched in pathways related to adipogenesis. And methylation changes at two specific loci persisted throughout the F1 and F2 generations [118]. In adult male zebrafish, prolonged exposure to DEHP (33–100 µg/L) caused increased gonadal DNA methylation that persisted into F1 offspring, hindering their development [17]. Additionally, 30 days of paternal DBP exposure (0.6 and 1.8 mg/L) induced developmental toxicity in F1 offspring, characterized by disrupted primordial germ cell (PGC) migration, inhibited spontaneous contractions, and persistent skeletal malformations in adulthood [119]. In conclusion, two interrelated pathways underlie transgenerational PAE toxicity in aquatic environments. First, a “chemical burden–developmental impairment” inheritance pattern is established when maternal PAE is transferred through internal exposure, which directly lowers offspring hatching success, survival, and reproductive fitness. Second, parental exposure causes germ cells to undergo persistent epigenetic changes, such as histone modifications, 5-hmC remodeling, and changes in DNA methylation. Paternal transmission is more frequently seen, and these heritable epigenetic changes subsequently disrupt the metabolic control, embryonic development, and reproductive signaling pathways of offspring. Both methods have so far been experimentally confirmed in a variety of aquatic species, such as fish and planktonic crustaceans (*D. magna*), showing remarkable concordance between molecular patterns and phenotypic results, cross-trophic conservation, and consistent low-dose effects. However, these harmful effects can spread and intensify over generations, endangering population stability and ecological integrity because aquatic creatures in natural settings frequently experience intergenerational exposure to PAE.

### 5.2. Population Degradation and Disturbance of Community Structure

In addition to directly harming individuals, prolonged exposure to PAEs has a negative influence on aquatic populations by lowering reproduction rates, changing the makeup of communities, and upsetting interspecies relationships. Population loss, a reduction in biodiversity, and a simplification of community organization are the eventual results of these consequences. DEHP dramatically reduced population tolerance thresholds by intensifying the reproductive and fatal impacts of marine heatwaves on sea urchin and copepod embryos at the population level [123]. Filter-feeding copepods (*Parvocalanus* and *Tigriopus*) exhibit synergistic suppression in their egg production, larval hatching, and population increase when microplastics co-occur with low concentrations of DEHP (0.1–0.3 μg/L) or DBP (10–100 μg/L) [124,125]. Remaining DMP concentrations in vertical flow-built wetlands that were continually dosed with 8.1 mg/L DMP for 114 days had a positive association with urease activity but a substantial negative correlation with microbial diversity. This showed that by modifying important enzyme activity and functional bacterial proportions, PAEs could reorganize microbial communities, impacting higher trophic levels and the integrity of aquatic food chains [126]. Additionally, the investigation showed that various biological groupings responded differently to PAE exposure. The populations of cladocerans, amphipods, and five representative taxa from tropical estuarine ecosystems (0.045–6 mg/L) drastically decreased after being exposed to DEHP, but microalgal–bacterial consortia displayed an increase in cell density. These results demonstrate notable variations in population tolerance to PAEs and metabolic compensation capabilities within ecosystem trophic levels [127]. In addition to reflecting the cumulative impacts of long-term pollution stress, the deterioration of population and community structure also suggests possible decreases in ecosystem performance.

### 5.3. Potential Cascading Effects on Ecosystems

PAEs have been shown to disturb community homeostasis and destabilize populations by affecting aquatic biota growth, development, reproduction, and metabolism. There are several ways in which these organism-level impacts could ripple down to the ecological level. First, PAEs prevent algae and phytoplankton from photosynthesizing. Aquatic primary productivity is ultimately reduced by these limitations, which also reduce algal growth rates and biomass [13,87,88,128]. Primary productivity limits resources for higher trophic levels because it maintains food-web energy transfer [129,130,131]. Second, decreases in zooplankton populations and PAE-driven bioaccumulation impair the effectiveness of energy transfer to higher consumers, lowering the biomass of top predators and the general functioning of the food web [69,124,125,127]. Third, PAEs lowered the population densities of important benthic species, reducing their ability to regulate phytoplankton, especially algae. Algal bloom risk may increase as a result of this loss of biological control, which could lead to an overabundance of phytoplankton populations [69,124,125,127]. Additionally, the reproductive function is disrupted by PAEs in fish, leading to reproductive deficiencies and transgenerational effects that ultimately jeopardize stock recruitment, endangering aquatic biodiversity and undermining fishing resources [76,77,78,98,108]. By reducing material cycling rates, energy flow efficiency, and vital biological control services through poison transmission at the individual, population, and community levels, PAEs collectively endanger aquatic ecosystems. Crucially, though, current research mostly concentrates on mechanisms at the individual level and lacks long-term field data to support models of population-scale community dynamics. To estimate ecological risks caused by PAEs, future research should develop multi-scale frameworks that integrate ecosystem-process models with species-interaction networks.

### 5.4. Molecular Pathway Responses and Associated Adverse Outcomes

**Figure 4:**
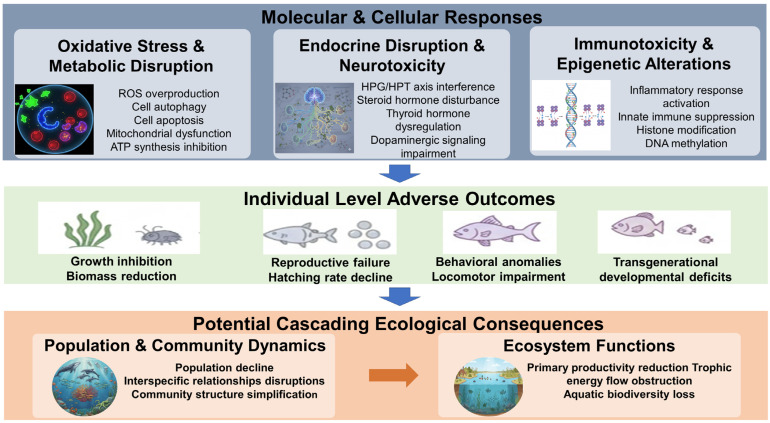
Conceptual framework of the multiscale toxicity cascade induced by PAEs in aquatic ecosystems: from molecular and cellular responses to potential ecological consequences.

Existing toxicological evidence elucidates that PAEs trigger cascading reactions by activating specific molecular pathways, ultimately precipitating significant morphological impairments and developmental deficits in aquatic organisms. There are several ways in which these organism-level impacts could ripple down to the ecological level (Table 2, Figure 4). First, the disruption of oxidative stress and energy metabolism pathways acts as a primary driver of growth inhibition and lethal effects. In primary producers, exposure to DMP and DEP induces an intracellular burst of reactive oxygen species (ROS) while inhibiting the activity of antioxidant enzymes (SOD, CAT) and the expression of photosynthetic pigment genes. This molecular-level oxidative damage directly precipitates lipid peroxidation (elevated MDA), cell autophagy/apoptosis and mitochondrial membrane potential collapse, resulting in growth stasis and biomass reduction in microalgae such as *P. tricornutum* and *C. vulgaris* [87,88]. In primary consumers like the rotifer *B. plicatilis*, BBP-induced mitochondrial damage blocks oxidative phosphorylation and hinders ATP synthesis; this molecular defect in energy metabolism manifests morphologically as reduced individual growth rates and diminished reproductive output [89].

Second, the dysregulation of endocrine and neuro-signaling pathways is directly linked to developmental malformations and reproductive decline. PAEs induce severe morphological consequences by interfering with gene expression along the HPG and HPT axes. For instance, chronic exposure to DBP and DEHP significantly downregulates the transcription of key steroidogenic genes (e.g., *cyp17a1*, *cyp19a*, *vtg*), leading to the suppression of sex hormone synthesis (E2, T). This molecular response translates directly into gonadal atrophy, arrested gametogenesis, and significantly reduced fertilization and hatching rates in adult zebrafish [106,107]. Concurrently, aberrant expression of thyroid hormone synthesis and transport genes (*tg*, *ttr*, *dio1/2*) disrupts T3/T4 homeostasis, causing developmental retardation such as reduced eye size and body length in Japanese medaka, *Oryzias latipes* [105]. Furthermore, alterations in neurotoxic molecular markers (e.g., *ache*, *drd1b*) are associated with the inhibition of dopamine synthesis, leading to behavioral anomalies including erratic swimming and impaired locomotor capacity [110,111,113].

Third, molecular toxicity propagates across generations through epigenetic inheritance and germline impairment. Mechanistic studies reveal that PAEs induce persistent DNA methylation marks that regulate gene expression in unexposed offspring. For example, zebrafish embryos exposed to MEHP exhibited differential methylation in adipogenic pathways (DMRs) that persisted into the F1 and F2 generations [118]. Similarly, paternal exposure to DBP has been directly linked to skeletal malformations and reduced heart rates in F1 offspring [119]. In invertebrates like water fleas, *D. magna*, DEHP-induced oxidative stress and gene dysregulation in the F0 generation resulted in cumulative declines in survival and net reproductive rates in subsequent generations [23].

Finally, these molecular-to-morphological damages at the individual level escalate into ecological consequences at the population level. The inhibition of algal growth compromises primary productivity, thereby limiting energy input into the entire food web. Meanwhile, high mortality rates, growth retardation, and reproductive failure in fish and invertebrates would directly result in population density declines and demographic instability. This cumulative damage across trophic levels threatens to simplify community structures and erode key ecosystem functions, such as biological control and nutrient cycling, posing a long-term threat to the sustainability of aquatic ecosystems.

## 6. Challenges and Future Directions

### 6.1. Gaps in Scientific Understanding

#### 6.1.1. Synergistic Toxicity Effects of PAEs and Other Plastic Additives

Little is known about the combined toxicity of PAEs and other plastic additives. Multiple plasticizers and stabilizers are frequently used in industrial production, which causes PAEs to coexist in aquatic ecosystems alongside microplastics, heavy metals, and other pollutants. Through electrostatic adsorption and hydrogen bonding, microplastics can concentrate PAEs, according to experimental findings, greatly increasing their toxicity and bioavailability [57]. However, the molecular-ecotoxicological mechanisms, metabolic transformation pathways, and accumulation kinetics of such complex pollutant combinations in aquatic species are not well understood. There is an urgent need for quantitative research on the synergistic impacts of several pollutants.

#### 6.1.2. Differences in Biological Responses Under Extreme Conditions

In aquatic animals, high salinity, high temperature, and hypoxia significantly change PAE uptake, transport, and excretion [123,132]. High temperatures speed up PAE leaching from plastics, boosting bioavailability; hypoxia may increase intake rates due to increased ventilation; and changes in salinity disturb ion control in fish gills, influencing PAE transmembrane flux. Further multidisciplinary research is needed to understand the molecular-physiological connection mechanisms underlying extreme environment–PAE co-stress.

#### 6.1.3. Cascading Effects of PAEs on Aquatic Ecosystems

Although research has shown that PAEs affect aquatic species’ ability to reproduce, develop, and behave, little is known about how they affect community structure, food-web energy flow, and ecosystem services. By influencing keystone species’ ability to survive and reproduce, PAEs have the potential to cause functional imbalances and restructuring of the food web. In order to systematically evaluate PAE hazards to ecosystem structure–function integrity, future research should combine multifactorial ecological models with long-term field observations.

### 6.2. Technological Innovation Needs

#### 6.2.1. Organoids and Multi-Organ Chips: Organ-Scale Toxicity Modeling

Promising instruments for simulating organ-scale toxicity are provided by organoid and multi-organ chip technology. The study of PAE biotransformation pathways and interorgan toxicity transmission is made possible by three-dimensional in vitro tissue models (organoids), which recreate the three-dimensional physiological structures of important detoxifying and barrier tissues (such as the liver and gills of fish). Additionally, dynamic monitoring of the transit of PAE metabolites across tissues is made possible by multi-organ chip systems. These methods increase the biological accuracy and physiological relevance of toxicity pathway forecasts while lowering the need for live animal testing.

#### 6.2.2. Cross-Scale Prediction Models: Bridging Risk Assessment from Individuals to Ecosystems

Responses at the population, community, and ecosystem levels may take much longer to appear in natural waterways, and ecological effects of low-concentration pollutants may only become apparent after extended exposure. Individual laboratory-based evaluations are the main focus of current PAE toxicity research. A multi-scale prediction framework that incorporates molecular toxicity measures, individual stress, population dynamics, and food-web architecture is desperately needed to bridge the gap between experimental data and actual ecosystem impacts. A prediction system that connects the molecular, individual, population, and ecosystem levels can be created by integrating physiological-toxicokinetic data into individual-based models and combining them with ecological network analysis and machine learning techniques. In order to promote adaptive environmental management, this integrated approach will be a crucial computational paradigm for converting lab results into ecological risk assessment.

Furthermore, traditional ecological risk assessments rely on apical endpoints, such as Median Effective Concentrations (EC_50_) or Predicted Effect Concentrations (PNECs), for mortality or growth inhibition. While this review highlights mechanistic toxicity pathways, a major barrier to regulatory application remains the gap between abundant molecular biomarker data and the apical endpoints required for risk assessment. Future research should prioritize developing quantitative adverse outcome pathways (qAOP), which mathematically link the magnitude of biomarker response to the probability of adverse outcomes. This “biomarker-to-risk” translation framework is essential for valorizing the massive amount of existing molecular data, converting them from theoretical observations into actionable environmental quality standards.

## Figures and Tables

**Table 1:** Bioaccumulation of PAEs in aquatic organisms at different trophic levels.

Species	Tissue	Bioaccumulation Concentration	Reference
Phytoplankton	Whole	DMP 68 ng/g ww; DEP 163 ng/g ww; DIBP 371 ng/g ww; DnBP 2302 ng/g ww; DHP 328 ng/g ww; DEHP 39,118 ng/g ww; DnOP 412 ng/g ww; DINP 58,762 ng/g ww	[32]
Phytoplankton	Whole	Σ_5_PAEs 1358 μg/g (DMP, DEP, DBP, BBP and DEHP)	[67]
Zooplankton	Whole	Σ_5_PAEs 72.7 μg/g (DMP, DEP, DBP, BBP and DEHP)	
Zooplankton	Whole	DMP 0–642 ng/g ww; DEP 7.0–162 ng/g ww; DnBP 38–1601 ng/g ww; DIBP 8.2–230 ng/g ww; BBP 0–340 ng/g ww; DEHP 309–43,228 ng/g ww; DnOP 0–3377 ng/g ww	[69]
*Mytilus galloprovincialis* (Mussel)	Soft tissue	DBBP 0.61 ng/g ww; DBP 26 ng/g ww; DEP 12 ng/g ww; DIBP 43 ng/g ww; DOP 0.07 ng/g ww; DMP 2.1 ng/g ww; DPP 0.093 ng/g ww	[80]
*Ctenopharyngodon idellus* (Grass carp)	Whole	DMP 14.78 ng/g ww; DEP 7.69 ng/g ww; DBP 148.71 ng/g ww; DEHP 100.32 ng/g ww	[76]
*Hypophthalmichthys molitrix* (Silver carp)	Whole	DMP 5.61 ng/g ww; DEP 6.51 ng/g ww; DBP 123.14 ng/g ww; DEHP 175.76 ng/g ww	
*Aristichthys nobilis* (Bighead carp)	Whole	DMP 9.20 ng/g ww; DEP 6.82 ng/g ww; DBP 117.12 ng/g ww; DEHP 131.67 ng/g ww	
*Mugil cephalus* (Flathead grey mullet)	Gill	DBP 100–3737 ng/g ww; DEHP 29–4107 ng/g ww; DEP 65–1221 ng/g ww; DINP 32–221 ng/g ww; DMP 179–369 ng/g ww; DnOP 42–260 ng/g ww	[60]
	Muscle	DBP 54–289 ng/g ww; DEHP 36–804 ng/g ww; DEP 41–224 ng/g ww; DINP 31–515 ng/g ww; DMP 178–195 ng/g ww; DnOP 39–123 ng/g ww	
*Carassius auratus* (Goldfish)	Whole	DMP 14.58 ng/g ww; DEP 11.05 ng/g ww; DBP 193.36 ng/g ww; DEHP 226.21 ng/g ww	[76]
*Cyprinus carpio* (Common carp)	Whole	DMP 12.93 ng/g ww; DEP 7.08 ng/g ww; DBP 110.64 ng/g ww; DEHP 131.04 ng/g ww	
*Hemiculter leucisculus* (Sharpbelly)	Whole	DMP 4.49 ng/g ww; DEP 12.19 ng/g ww; DBP 66.94 ng/g ww; DEHP 100.45 ng/g ww	
*Sardina pilchardus* (Sardine)	Whole	DMP 0.633 ng/g ww; DEP 2.35 ng/g ww; DIBP 47.8 ng/g ww; DBP 100 ng/g ww; BBP 1.88 ng/g ww; DEHP 57.0 ng/g ww; DINP 9.10 ng/g ww; DNOP 6.95 ng/g ww	[56]
*Boops boops* (Bogue)	Whole	DMP 0.398 ng/g ww; DEP 1.58 ng/g ww; DIBP 15.1 ng/g ww; DBP 30.4 ng/g ww; BBP 1.42 ng/g ww; DEHP 89.7 ng/g ww; DINP 2.39 ng/g ww; DNOP 2.70 ng/g ww	
*Merluccius merluccius* (European hake)	Whole	DMP 0.131 ng/g ww; DEP 3.46 ng/g ww; DIBP 24.5 ng/g ww; DBP 17.2 ng/g ww; BBP 1.16 ng/g ww; DEHP 36.6 ng/g ww; DINP 1.32 ng/g ww	
*Mullus barbatus* (Red mullet)	Gill	DBP 97–680 ng/g ww; DEHP 134–542 ng/g ww; DEP 87–411 ng/g ww; DINP 76–6601 ng/g ww; DMP 181–1273 ng/g ww; DnOP 80–6523 ng/g ww	[60]
	Muscle	DBP 64–141 ng/g ww; DEHP 18–6739 ng/g ww; DEP 65–204 ng/g ww; DINP 31–488 ng/g ww; DMP 178–275 ng/g ww; DnOP 39–482 ng/g ww	
	Whole	DMP 0.388 ng/g ww; DEP 7.69 ng/g ww; DAP 5.55 ng/g ww; DIBP 176 ng/g ww; DBP 142 ng/g ww; BBP 3.10 ng/g ww; DEHP 107 ng/g ww; DINP 10.4 ng/g ww; DNOP 6.08 ng/g ww	[56]
*Diplodus annularis* (Annular seabream)	Gill	DBP 54–827 ng/g ww; DEHP 11–2689 ng/g ww; DEP 57–257 ng/g ww; DINP 48–698 ng/g ww; DMP 192–1293 ng/g ww; DnOP 56–830 ng/g ww	[60]
	Muscle	DBP 59–246 ng/g ww; DEHP 30–636 ng/g ww; DEP 49–141 ng/g ww; DINP 35–687 ng/g ww; DMP 178–453 ng/g ww; DnOP 39–838 ng/g ww	
*Sardina pilchardus* (Sardine)	Whole	DMP 0.779 ng/g ww; DEP 2.62 ng/g ww; DIBP 23.9 ng/g ww; DBP 29.3 ng/g ww; BBP 2.15 ng/g ww; DEHP 69.7 ng/g ww; DINP 18.7 ng/g ww; DNOP 7.52 ng/g ww	[56]
*Boops boops* (Bogue)	Whole	DMP 0.201 ng/g ww; DEP 3.42 ng/g ww; DIBP 14.3 ng/g ww; DBP 12.2 ng/g ww; BBP 5.14 ng/g ww; DEHP 93.1 ng/g ww; DINP 4.05 ng/g ww; DNOP 7.78 ng/g ww	
*Mullus barbatus* (Mullus barbatus)	Whole	DMP 0.539 ng/g ww; DEP 3.42 ng/g ww; DIBP 14.3 ng/g ww; DBP 12.2 ng/g ww; BBP 5.14 ng/g ww; DEHP 93.1ng/g ww; DINP 4.05 ng/g ww; DNOP 7.78 ng/g ww	
*Merluccius merluccius* (European hake)	Whole	DMP 0.0452 ng/g ww; DEP 1.47 ng/g ww; DAP 0.0966 ng/g ww; DIBP 2.36 ng/g ww; DBP 7.38 ng/g ww; BBP 4.23 ng/g ww; DEHP 78.3 ng/g ww; DINP 1.36 ng/g ww; DNOP 0.731 ng/g ww	
*Engraulis encrasicolus* (European anchovy)	Whole	DEHP 17.6 ng/g ww; DIDP 11.1 ng/g ww; DINP 8.8 ng/g ww; DBP 8.9 ng/g ww	[78]
*Sardina pilchardus* (Sardine)	Whole	DEHP 19.4 ng/g ww; DIDP 11.2 ng/g ww; DBP 9.9 ng/g ww	
*Boops boops* (Bogue)	Whole	DEHP 16.6 ng/g ww; DIDP 12.6 ng/g ww; DBP 13.4 ng/g ww; DBP 8.8 ng/g ww; MNBP 9.8 ng/g ww	
*Mullus barbatus* (Red mullet)	Whole	DEHP 13.7 ng/g ww; DIDP 11.7 ng/g ww; DBP 8.2 ng/g ww	
*Engraulis encrasicolus* (European anchovy)	Whole	DEHP 15.4 ng/g ww; DIDP 12.2 ng/g ww; DBP 9.9 ng/g ww	
*Sardina pilchardus* (Sardine)	Whole	DEHP 14.8 ng/g ww; DIDP 11.3 ng/g ww; DBP 8.3 ng/g ww	
*Boops boops* (Bogue)	Whole	DEHP 16.2 ng/g ww	
*Mullus barbatus* (Red mullet)	Whole	DEHP 17.4 ng/g ww; DIDP 10.1 ng/g ww; DBP 9.9 ng/g ww	
	Muscle	Σ_6_PAEs 0.60–3.55 mg/kg dw (DMP, DEP, DBP, DIBP, DEHP and DINP)	[77]
*Pelteobagrus fulvidraco* (Yellow catfish)	Whole	DMP 29.42 ng/g ww; DEP 11.45 ng/g ww; DBP 174.90 ng/g ww; DEHP 358.32 ng/g ww	[76]
*Culter alburnus* (Culter)	Whole	DMP 15.61 ng/g ww; DEP 8.38 ng/g ww; DBP 184.33 ng/g ww; DEHP 211.60 ng/g ww	
*Silurus asotus* (Amur catfish)	Whole	DMP 9.01 ng/g ww; DEP 7.82 ng/g ww; DBP 146.53 ng/g ww; DEHP 239.14 ng/g ww	
*Siniperca chuatsi* (Mandarin fish)	Whole	DMP 3.74 ng/g ww; DEP 4.71 ng/g ww; DBP 67.51 ng/g ww; DEHP 160.97 ng/g ww	
*Channa argus* (Northern snakehead)	Whole	DMP 7.02 ng/g ww; DEP 4.67 ng/g ww; DBP 75.86 ng/g ww; DEHP 72.48 ng/g ww	
*Thunnus albacares* (Yellowfin tuna)	Red meat	DMP 3.25–81.64 ng/g ww; DEP 5.76–89.40 ng/g ww; DIBP 22.09–143.23 ng/g ww; DnBP 250.85–6851.12 ng/g ww; DHP 1.56–349.46 ng/g ww; BBP 71.91–5681.92 ng/g ww; DEHP 54.47–436.13 ng/g ww; DnOP 124.02–3579.88 ng/g ww; DINP 1751.03–94,221.7 ng/g ww; DiDP 38.14–1745.19 ng/g ww	[63]
	White meat	DMP 0.67–2.02 ng/g ww; DEP 3.38–8.83 ng/g ww; DIBP 6.78–15.50 ng/g ww; DnBP 31.24–355.56 ng/g ww; DHP 0.36–6.45 ng/g ww; BBP 2.98–261.14 ng/g ww; DEHP 33.60–495.23 ng/g ww; DnOP 0.91–62.66 ng/g ww; DINP 20.13–205.10 ng/g ww; DiDP 3.34–57.21 ng/g ww	
Fish	Muscle	Σ_8_PAEs 465 ng/g	[81]
Shrimp	Muscle	Σ_8_PAEs 293 ng/g	
Crab	Muscle	Σ_8_PAEs 811 ng/g	
Shellfish	Muscle	Σ_8_PAEs 261 ng/g	

**Table 2:** Toxicological effects of PAEs on aquatic organisms at the individual level.

Toxicity Type	Organism	Chemical	Exposure (Conc./Time)	Toxic Effects	Reference
Oxidative stress	*Phaeodactylum tricornutum*	DMP	50, 100, 200, 400 mg·L−1/96 h	ROS ↑; Growth ↓; SOD ↑; POD ↑; Maximum quantum yield (F_v_/F_m_) ↓	[87]
		DEP	400 mg·L−1/96 h	ROS ↑; Growth ↓; SOD ↑; POD ↑; F_v_/F_m_ ↓;	
	*Chlorella vulgaris*	DEHP	2, 4, 6, 8, 10 mg·L−1/5 d	H_2_O_2_ ↑; MDA ↑; GPx ↓	[88]
	*Brachionus plicatilis*	BBP	0.001, 0.01, 0.10, 1.00 mg·L−1/48 h	SOD ↑; CAT ↓; GSH ↓; MDA ↑; *hsp70* ↑	[89]
	*Daphnia magna*	DBP	100 μg·L−1/21 d	SOD ↑; CAT ↑; GST ↑; MDA ↑	[90]
		BBP	100 μg·L−1/21 d	SOD ↑; CAT ↑; GST ↑; MDA ↑	
	*Mytilus coruscus*	DEHP	0.04, 0.40, 1.00 mg·L−1/28 d	SOD ↑; CAT ↑; GSH ↑	[52]
		DBP	0.04, 0.40, 1.00 mg·L−1/28 d	SOD ↑; CAT ↑; GSH ↑	
	*Ruditapes philippinarum*	DEHP	50 μg·L−1/48 h	SOD ↓; CAT ↓; ROS↑; MDA ↑	[91]
	*Tegillarca granosa*	DEHP	50 μg·L−1/48 h	SOD ↑; CAT ↑; ROS↑; MDA ↑; POD ↓	
	*Portunus trituberculatus*	DBP	0.2, 2, 10 mg·L−1/13 d	MDA ↑; *hsp70* ↑; *hsp90* ↑; SOD ↓; CAT ↓	[92]
	*Carassius auratus*	DEHP	20, 100, 500 μg·L−1/96 h	ROS ↑; MDA ↑; SOD ↑; CAT ↑; GSH-PX ↑; GSH ↓	[93]
	*Danio rerio*	DBP	250, 500, 1000 μg·L−1/28 d	SOD ↓; CAT ↓; TAC ↓; MDA ↑; *sod* ↓; *cat* ↓; and *nrf2* ↓	[94]
Metabolic disruption	*Phaeodactylum tricornutum*	DMP	50, 100, 200, 400 mg·L−1/96 h	Soluble protein ↑; F_v_/F_m_ ↓	[87]
	*Spirodela polyrhiza*	DMP	3, 30, 600 mg·L−1/7 d	Total chlorophyll ↓; Photosynthetic electron transport rate through PSII (ETR) ↓; Effective quantum yield ↓; Growth Metabolism ↓	[13]
	*Daphnia magna*	DBP	100 μg·L−1/21 d	TCA intermediates ↓; Oxoglutaric acid ↓; Metabolites ↓	[90]
	*Brachionus plicatilis*	BBP	001, 0.01, 0.10, 1.00 mg·L−1/48 h	Oxidative phosphorylation; TCA, Glycolysis ↓	[89]
	*Mytilus coruscus*	DBP	0.04, 0.40, 1.00 mg·L−1/28 d	Palmitic acid ↑; γ-linolenic acid ↓	[52]
		DEHP	200 μg·L−1/15 d	Palmitic acid and stearic acid ↓; Glutamate, aspartate, alanine, and proline ↓	[95]
	*Mytilus galloprovincialis*	DEHP	4, 12, 36, 108, 324 μg·L−1/14 d	Glucose, glycogen ↑ (12μg/L); Glucose and glycogen ↓ (324μg/L); Amino acids ↓ (12μg/L); Amino acids ↑ (324μg/L); Homarine ↑	[96]
	*Penaeus vannamei*	DEHP	10, 100, 1000 μg·L−1/21 d	*ade5* and *uro* ↑; *pde3b* and *enpp4* ↓	[97]
	*Clarias gariepinus*	DEHP	10, 100, 200, 400 μg·L−1; 14 d	E2 in females ↑; T in males ↑; *vtg* ↑; er-α ↑; *cyp19a1* ↑	[98]
	*Danio rerio*	DINP	0.042, 0.42, 4.2, 42 μg·L−1/21 d	Lipid vacuole area ↑; FT-IR ↑	[99]
		DEHP	Overfed (20 mg/fish/day) + 3 mg DEHP/kg, 60 d	Body mass, hepatosomatic and gonadosomatic indices↑; *dgat2* ↓; *acox-3* ↓; *hnf4a* ↓; DEHP-driven effects on obesity	[100]
	*Sparus aurata*	DINP	15, 1500 μg·kg−1 bw diet/21 d	11-KT ↓; E2 ↑	[101]
	*Pelteobagrus fulvidraco*	DEHP	0.1, 0.5 mg·L−1/56 d	GLU ↑; TG ↑; ALT ↑; AST ↑; HSI ↓	[102]
Thyroid disruption	*Danio rerio*	DBP	1, 5, 10, 50 μg·L−1/96 h	T3 ↑; T4 ↑; *tg* ↑; *ttr* ↑; *dio2* ↑	[103]
		DIBP	1, 10, 50, 100 μg·L−1/96 h	T3 ↑; T4 ↑; *tg* ↑; *ttr* ↑; *dio2* ↑	
		DINP	0.3, 1.0, 3.0, 6.0 mg·L−1/5 d	TT4 ↑; fT_3_ ↑; TSH ↑; *tshβ* ↑; *tshr* ↑; *dio2* ↑	[104]
	*Oryzias latipes*	DEHP	32, 100, 320, 1000 μg·L−1/9 d	*tshβ*-like ↑; *tshβ* ↑; *dio1* ↑; *dio2* ↑; *trα* ↑; *trβ* ↑; Eye size ↓; Body length ↓	[105]
Reproductive toxicity	*Danio rerio*	DEHP	10, 33, 100 μg·L−1/90 d	T ↑; E2 ↑; *cyp17a1* ↓; *hsd17b3* ↓; *cyp19a*↑; Fertilization rate ↓	[17]
			100 μg·L^−1^/111 d	Sex ratio of females vs. males ↑; Fertilization rate ↓; Percentage of spermatocytes ↓; Disruption of progesterone receptor signaling pathway	
	*Danio rerio*	DEP	6, 12.5, 25.0 mg·L−1/120 d	GSI↓; Atretic follicles ↑; Offspring survival ↓; VTG ↓	[106]
		DINP	10 mg·L−1/210 d		
		DEHP	10 mg·L−1/210 d		
	*Danio rerio*	DBP	4.9, 13.6, 43.8 μg·L−1 until sexual maturation	Fecundity ↓; GSI ↑; E2 ↓; T ↑; Percentage of vitellogenic oocytes ↑; Percentage of spermatocytes ↑; *cyp19a* ↓; cyp17 ↑	[107]
	*Cyprinus carpio*	DEHP	1, 10, 100 μg·L−1/60 d	GSI ↓; Fecundity ↓; *cyp19a1a* ↓; *cyp17a1*, *fsh\beta*, *lh\beta*, *esr1* and *esr2a* ↓	[108]
Neurotoxicity	*Daphnia magna*	DEP	4.5, 45 mg·L−1/48 h	GABA ↓; Adenosine ↑; Isoleucine ↓; Caenitine and guanosine ↓	[109]
	*Mytilus coruscus*	DEHP	200 μg·L−1/15 d	Choline ↓; GABA ↓	[95]
	*Danio rerio*	DINP/DEHP	0.5–10,000 μg·L−1/120 h	DINP: *ache* and *drd1b* ↑; DEHP: *ache* ↑, *drd1b* ↓	[110]
		DEHP	1, 2.5, 5, 10 mg·L−1/7 d	Locomotor activity↓; Dopamine Content ↓; Dopamine synthesis, reuptake and metabolism related genes (*th*, *dat*, *mao*) ↓	[111]
		DEHP	50 μM/21 d	Light preference ↑; Neuronal pyknosis ↑; Chromatin condensation ↑; Oxidative stress in brain ↑	[112]
		MEHP	20, 200, 800 μg·L−1/120 hpf	Oxidative stress and cell apoptosis in brain↑; Swimming activity, aberrant social behavior, and locomotor capacity ↓; Nervous system-related genes (*vamp1* and *dnm1b* ↑, *slc6a11a* ↓)	[113]
Immunotoxicity	*Mytilus galloprovincialis*	DEHP	4, 12, 36, 108, 324 μg·L−1/14 d	*nf-κb* ↑, *hsp70* ↓; *MgGLYZ* ↓ (12 μg·L−1)	[96]
	*Macrophthalmus japonicus*	DEHP	1, 10, 30 μg·L−1/7 d	PO ↓; proPO and LGBP ↓; Serpin ↑	[114]
	*Danio rerio*	DEHP	1, 2, 5 mg·L−1/96 h	C3 ↑; Complement C3 pathway biomarker genes (*c3ar1*, *c7a*, *c5ar1*, *c1qa*, *c9*, *masp2* ↑)	[19]
		DBP	0.05, 0.5, 50, 500 μg·L−1/5 dpf	Chronic inflammation response ↑; HPA axis function ↓	[115]
	*Labeo catla*	DEP	1.62 mg·L−1/30 d	*myd88* and *igm-α* ↑	[116]
Transgenerational toxicity	*Daphnia magna*	DEHP	F0: 1 μg·L−1/until spawning begins	Reactive oxygen species in F0–F2 ↑; Fat deposition in F0–F3 ↑; DEHP accumulation from F0 to F1–F3	[23]
	*Caretta caretta*	DBP, DEHP, DEP, DOTP	Sample on Linosa Island	PAE maternal yolk transfer	[117]
	*Danio rerio*	MEHP	F0: 30 μM/6 dpf	DMRs transgenerationally inherited from F0 to F1–F2	[118]
		DBP	F0: 0.2, 0.6, 1.8 mg·L−1/30 d	Malformation rate and heart rate in F1 ↑; Primordial germ-cell migration disruption and skeletal malformations in F1	[119]
		DEHP	F0: 10, 33, 100 μg·L^−1^/90 d	Gonadal DNA methylation level in F0–F1 ↑; Body weight in F1 ↓	[17]

## Data Availability

No new data were created or analyzed in this study. Data sharing is not applicable to this article.

## Abbreviations

AOP, adverse outcome pathway; BAF, bioaccumulation factor; BBP, butyl benzyl phthalate; BCF, bioconcentration factor; BMF, biomagnification factor; CAT, catalase; DBP, dibutyl phthalate; DEHP, di(2-ethylhexyl) phthalate; DEP, diethyl phthalate; dpf, days post-fertilization; dw, dry weight; ER, estrogen receptor; GPx, glutathione peroxidase; GSH, glutathione; GST, glutathione S-transferase; H2O2, hydrogen peroxide; HMW, high molecular weight; HPG axis, hypothalamic–pituitary–gonadal axis; Koc, organic carbon–water partition coefficient; Kow, octanol–water partition coefficient; LMW, low molecular weight; MDA, malondialdehyde; MIE, molecular initiating event; MPs, microplastics; PAEs, phthalate esters; PPARs, peroxisome proliferator-activated receptors; PVC, polyvinyl chloride; qAOPs, quantitative adverse outcome pathways; ROS, reactive oxygen species; SOD, superoxide dismutase; VTG, vitellogenin; ΣPAEs, sum of phthalate esters.
